# Modern Sociology

**Published:** 1900-01-13

**Authors:** 


### Modern Sociology.

### HEALTH AND EDUCATION.

THOUGH the cry that our children are suffering from over-pressure and cram through the education they are compelled to accept is now an old one, it does not seem to become so stale as one would expect in the ears of the public. Whether the repetition implies a real interest in the children of the people, or a dislike to the education of the masses, is not for us to inquire here; but it is worth while to ask if it is indeed true that the health of our youth is being sacrificed to their mental development. What are the facts? Do more children die during school-life now than formerly; or rather, to speak more accurately, is the mortality of children greater now during the period they are at school than it was during the same age-period at a time when they did not attend school? Some information on the point may be gained from a pamphlet by Dr. A. K. Chalmers, medical officer of health for Glasgow, entitled "An Inquiry into the Vital Statistics of School Ages." That Dr. Chalmers's figures deal for the most part only with Scotland does not vitiate them for such an inquiry, as Scotland has, on the whole, taken more heartily to education than England.

Dr. Chalmers takes school age to be the period between five and 15. This is, of course, only approximately accurate. Some children leave school before they are 15, others remain till they are older. The latter are, however, comparatively few. Though a large number of the children of the poor leave school before they are 15, the 15 limit gives the opportunity of including in the death-rate the fatal issues of any ailment contracted during school-life, while excluding those ailments in which sex is the predominating factor. The time between five and 15 seems, on investigation, to be the healthiest of human life, the period when the resistance to disease is strongest. The returns of the Registrar-General show that in Scotland, in 1896, the death-rate of young persons was as follows:—

| | |
|---|---|
| Under 5 ... ... ... | 49·2 per thousand |
| 5-10 ... ... ... ... | 4·4 ,, ,, |
| 10-15 ... ... ... ... | 3·1 ,, ,, |
| 15-20 ... ... ... ... | 4·9 ,, ,, |

But has this vigour, this power of resisting disease, been lessened in any way by the spread of education? An answer to that question may be found in the following table, which gives the number of deaths per million inhabitants of Scotland of the ages between five and 15 during certain years, 10 years apart from each other:—

| | |
|---|---|
| Years 1860-1862 ... ... | 7,699 per million |
| ,, 1870-1872 ... ... | 7,689 ,, ,, |
| ,, 1880-1882 ... ... | 6,097 ,, ,, |
| ,, 1890-1892 ... ... | 4,847 ,, ,, |

As, however, the death-rate for all ages has decreased during the last 30 years, owing to our greater knowledge of the laws of health, and consequent improved sanitation, the diminution in the mortality of children of school age may be set down to this cause alone. Certainly there has been a decrease in the general average death-rate, and a very marked one in the case of children under five years of age, who always give the largest proportion of deaths. Thus, in 1862 the death-rate of children under five was 62,597 per million, in 1870 it was 59,670, and in 1890 it had fallen to 52,437 per million. But taking the years 1870-72 and 1890-92, that is, the period from the passing of the Education Act until the last census, we find that the reduction in the death-rate stands thus:—

| | |
|---|---|
| Under 5 ... ... ... ... | 12 per cent. |
| 5-15 ... ... ... ... ... ... | 38 ,, ,, |

So the gain in young lives saved is even greater in the years of school-life than it is in childhood, and it is also incomparably greater than in later years. There are certainly many reasons, not strictly educational, why this improvement should be. It must be remembered that the bulk of the children who are now compelled to go to school do not belong to the favoured classes. Formerly they were permitted to go to work at an earlier age than now, and the hours and conditions of work were not as carefully defined and limited as they are to-day. The advantage of the Education Act in this respect is very real, though only indirect. Moreover, the average school, especially now when so much attention is paid to school architecture, is a healthier place than many a home. The rooms are clean, well ventilated, well warmed. It is better for a child's health that he should be in school than in the slum or the street. In school, too, the child of destitute or even unworthy parents comes in contact with various charitable agencies, and is often better fed and clothed than he would otherwise be. Looking at the matter quite apart from education itself it can hardly be doubted that the massing of the children of the poor before an army of not unsympathetic critics, in the form of teachers, helps to bring their necessities before the public, and greatly facilitates the giving of assistance. In this way the provisions of the Education Act make for the health of youth.

It might be thought, however, that this same massing of the children would contribute to the spread of infection. In this idea there is some truth. But the spread of a disease in a school is much more promptly noted than a similar spread of disease in a locality. If one child after another is kept away because of measles—which is the chief epidemic to be found in schools—the teacher, anxious about his attendances and his Government grant, is much more willing to notify the medical officer and get his school closed than the average parent is to avow the fact of the existence of infectious disease in his home. And it is worth noting in regard to this, that in Scotland the percentage of deaths from zymotic disease between the ages 5-15 fell from 3·3 per thousand in 1870-1872 to 1·3 in 1890-1892. In the death-rate from tuberculous diseases there has not been anything like the same improvement as in zymotics, though Dr. Chalmers reckons it at 700 per million of lives between five and 15 in 1890-1892 as compared with the same period 20 years previous. But the decrease in the deaths from these ailments is greater both below and above school age. Apart from the fact, however, that the 10 years between five and 15 are the most vigorous of life, we must remember that tuberculous diseases oftenest claim their victims in early maturity, even though the seeds are sown earlier; and perhaps the better atmosphere given by the modern school may deserve some credit for the 1,300 per million reduction in deaths from these causes at ages over 15.

